# Impact of serum sodium concentrations, and effect modifiers on mortality in the Irish Health System

**DOI:** 10.1186/s12882-023-03251-w

**Published:** 2023-07-06

**Authors:** Conor Walsh, Leonard D. Browne, Robert Gilligan, Rose Galvin, Liam Glynn, Cathal Walsh, Austin G. Stack

**Affiliations:** 1 School of Medicine, University of Limerick, Limerick, Ireland; 2 Health Research Institute (HRI), University of Limerick, Limerick, Ireland; 3 Department of Nephrology, University Hospital Limerick, St Nessans Rd, Limerick, Ireland; 4 School of Allied Health, University of Limerick, Limerick, Ireland; 5 Department of Mathematics and Statistics, University of Limerick, Limerick, Ireland

**Keywords:** Mortality, Kidney disease, Sodium, Dysnatraemia

## Abstract

**Background:**

Abnormalities of serum sodium are associated with increased mortality risk in hospitalised patients, but it is unclear whether, and to what extent other factors influence this relationship. We investigated the impact of dysnatraemia on total and cause-specific mortality in the Irish health system while exploring the concurrent impact of age, kidney function and designated clinical work-based settings.

**Methods:**

A retrospective cohort study of 32,666 participants was conducted using data from the National Kidney Disease Surveillance System. Hyponatraemia was defined as < 135 mmol/L and hypernatraemia as > 145 mmol/L with normal range 135–145 mmol/L. Multivariable Cox proportional hazards regression was used to estimate hazard ratios (HR’s) and 95% Confidence Intervals (CIs) while penalised spline models further examined patterns of risk.

**Results:**

There were 5,114 deaths (15.7%) over a median follow up of 5.5 years. Dysnatraemia was present in 8.5% of patients overall. In multivariable analysis, both baseline and time-dependent serum sodium concentrations exhibited a U-shaped association with mortality. Hyponatremia was significantly associated with increased risk for cardiovascular [HR 1.38 (1.18–1.61)], malignant [HR: 2.49 (2.23–2.78)] and non-cardiovascular/non-malignant causes of death [1.36 (1.17–1.58)], while hypernatremia was significantly associated with cardiovascular [HR: 2.16 (1.58–2.96)] and non-cardiovascular/ non-malignant deaths respectively [HR: 3.60 (2.87–4.52)]. The sodium-mortality relationship was significantly influenced by age, level of kidney function and the clinical setting at baseline (*P* < 0.001). For hyponatraemia, relative mortality risks were significantly higher for younger patients (interaction term *P* < 0.001), for patients with better kidney function, and for patients attending general practice [HR 2.70 (2.15–3.36)] than other clinical settings. For hypernatraemia, age and kidney function remained significant effect modifiers, with patients attending outpatient departments experiencing the greatest risk [HR 9.84 (4.88–18.62)] than patients who attended other clinical locations. Optimal serum sodium thresholds for mortality varied by level of kidney function with a flattening of mortality curve observed for patients with poorer kidney function.

**Conclusion:**

Serum sodium concentrations outside the standard normal range adversly impact mortality and are associated with specific causes of death. The thresholds at which these risks appear to vary by age, level of kidney function, and are modified in specific clinical settings within the health system.

**Supplementary Information:**

The online version contains supplementary material available at 10.1186/s12882-023-03251-w.

## Introduction

Abnormalities of serum sodium, or dysnatraemia, are some of the most common electrolyte abnormalities observed among community and hospitalised populations [1–5]. An increasing body of evidence has linked these abnormalities with increased mortality, morbidity, and length of stay in diverse patient cohorts [3–8]. Hyponatremia, defined as a serum sodium level < 135 mmol/L, is caused by disequilibrium between body salt and water, either from loss of body salt or from relative or absolute excess of body water, and is associated with heart failure, liver disease, acute kidney injury, malignancy, and intracerebral haemorrhage [6, 7]. Hypernatraemia, defined as a serum sodium level > 145 mmol/L, occurs far less frequently than hyponatraemia but is an equally serious condition [8–10]. Observational studies have suggested that serum sodium exhibits a U-shaped association with mortality with threshold of risks that begin even within the normal clinical range of serum sodium concentration [2, 3]. While most studies of hypo- and hypernatremia have focused on hospitalised patients or in the elderly, few have addressed the impact of dysnatraemia on mortality across the age spectrum, by level of kidney function, and within designated settings across the health system.

The risk of developing both hyper-and hyponatraemia and their attendant consequences may be far greater for some patients than others and may vary according to clinical settings and underlying health state. For example, recent studies have revealed that dysnatraemia is common in patients with CKD and predicts higher death rates [11–13]. In a large cohort of US veterans with CKD not requiring dialysis, Kovesdy et al. revealed a U-shaped association between serum sodium concentrations and mortality independent of coexisting medical illness [13]. Interestingly, the mortality risks were attenuated for patients with more severe kidney impairment. Han et al. also reported higher mortality risks for hypo- and hypernatraemia in a prospective cohort of CKD patients but with higher than expected mortality risk for patients even in the “low normal” serum sodium range of 135–140 mmol/L [12]. These observations have raised suspicions as whether there is better tolerance of dysnatraemia in patients with progressive CKD, and whether the threshold values for survival vary by GFR, although patients with CKD are hypothesised to be more susceptible to development of dysnatraemia as a result of progressively diminished renal concentrating capacity [1]. Recent evidence suggests that the mortality risks from dyskalaemia are less impactful in CKD due to greater adaptive mechanisms [14]. One might speculate that that the same holds true for dysnatraemia but the evidence to support such an assuption is scant. Similarly, it remains to be proven whether other factors such as age, and designated clinical setting at baseline might also influence the sodium-mortality relationships and specific mortality thresholds.

The principal goals of this study were to: describe the nature and impact of serum sodium on all-cause and cause-specific mortality among patients in a large integrated health system; to determine the impact of patient age and level of kidney function on sodium-mortality thresholds, and to explore whether the clinical setting at baseline within the health system modified the strength and shape of the sodium-mortality association.

## Methods

### Data source

The National Kidney Disease Surveillance System (NKDSS) served as the primary data source for this observational study [15, 16]. The NKDSS monitors the health and outcomes of patients with kidney disease in the Irish health system. The principal data sources included regional laboratory information systems, which capture a comprehensive list of laboratory results from inpatients and outpatients within a designated health region; dialysis registers which capture clinical data on patients who progress to End Stage Kidney Disease (ESKD); and mortality data files from the national Central Statistics Office (CSO). The final merged dataset captured information on demographic characteristics, county of residence, clinical location of blood test capture (clincal setting), multiple laboratory measures of health status, dialysis indicator variables and death.

### Cohort creation

All patients with a valid renal profile blood sample (including serum creatinine, plasma urea, serum potassium, serum sodium, serum chloride, plasma bicarbonate, serum phosphate, serum calcium, and serum albumin) in the Midwest region in the Republic of Ireland from 1/1/2007 to 31/12/2012 were included. The analysis was restricted to patients over 18 years or older with valid serum sodium measurements and concurrent serum glucose measurements with vital status through to 31/12/2013. Patients receiving dialysis (haemodialysis and peritoneal dialysis) were excluded and this resulted in a final cohort of 32,666 patients. All patients were followed up until death or the end of data collection 31/12/2013.

### Exposure

Serum sodium recorded at first measurement for each patient within the health system was used for the baseline serum sodium analysis. For each serum sodium, the concomitant plasma glucose concentrations was included for adjustment. Serum sodium was corrected for glucose levels > 11.1 mmol/L (> 200 mg/dL) by the equation developed by Hillier et al. [17].

$$\mathrm{Corrected\; Serum\; Sodium}=\mathrm{Serum\; Sodium}+2.4*\left(\frac{\mathrm{glucose\;}\left(\mathrm{mmol}/\mathrm{L}\right)-100}{100}\right)$$

Hyponatremia was defined as < 135 mmol/L and hypernatremia was defined as > 145 mmol/L. For time-dependent repeated measures analysis, we included baseline serum sodium concentrations as well as all serum sodium concentrations measured during the follow-up. We used carry-forward values to fill in data for months where the serum sodium data were not available.

### Covariates

Data was captured on age, sex, designated clinical setting at baseline, and an extensive range of blood biomarkers which indicated the presence and severity of clinical disease. Plasma values of alanine aminotransferase (ALT) and alkaline phosphatase (ALP) were markers of liver function. Haemoglobin values served to capture the presence of anaemia while serum albumin served as a marker of nutrition and inflammation. The white blood cell (WBC) count served as a marker of inflammation. Lastly, plasma urea and serum creatinine concentrations were used to assess kidney function. Serum creatinine levels were used to determine the estimated glomerular filtration rate (eGFR) in ml/min per 1.73m^2^ based on the Chronic Kidney Disease Epidemiology Collaboration (CKD-EPI) [18]. The clinical setting at baseline was defined as the location where the creatinine test was ordered by the supervising physician at recruitment and categorised as inpatient facility (IP), outpatient facility (OP), general practice (GP) and emergency room (ER). The date of death and primary cause of death were determined from national mortality files provided by the Central Statistics Office (CSO).

### Outcomes

Mortality data was ascertained from national mortality data files from the Central Statistics Office. The underlying cause of death was coded according to the International Classification of Diseases, Tenth Revision (ICD-10). We classified deaths into three major categories: (i) cardiovascular deaths [ICD-10: I00-I99], (ii) malignancy-related deaths [ICD-10: C00-C97, D00-D48] and (iii) non-cardiovascular/non-malignancy-related deaths.

### Statistical analysis

Baseline characteristics of all subjects, by category were summarised, and described using percentages for categorical data, mean and standard deviation for continuous variables or median and interquartile range for continuous variables with skewed distribution. Group comparisons for continuous variables were performed using the Kruskal Wallis test while group comparisons for categorical variables were performed with the Chi-square test. Crude and age adjusted mortality rates were estimated with poission regression by strata of serum sodium and were expressed as deaths per 1,000 person-years. Cox proportional hazard regression was used to model relationships of serum sodium with all-cause and cause-specific mortality adjusting for baseline characteristics. Covariates for adjustment included age, sex and core laboratory health indicators measured concurrently at baseline. Associations of serum sodium with mortality were expressed as hazard ratios (HR) with 95% Confidence Intervals (CI) in univariate and multivariable models. To acocunt for repeated measures of corrected sodium during the follow up period, the association between serum sodium levels and mortality was further examined in time-dependent Cox models.

To further examine the time-dependent associations between serum sodium concentrations and mortality, we fitted penalised splines models for sodium values where the optimal spline smoothing parameter was selected by minimising the Akaike information criterion (AIC). These splines allowed us to model a flexible non-linear relationship between sodium and mortality. Effect modification was explored by including cross-product interaction terms in the Cox models that examined the impact of baseline covariates on the serum sodium-mortality relationship. Effect modification was tested for age, eGFR, CKD as defined as eGFR threshold of less than 60 ml/min/1.73m^2^, and clinical setting at baseline on the sodium-mortality relationship. We also tested for the presence of these same interactions in the time-dependent Cox models. All statistical analyses were performed using R (version 4.0; http://www.r-project.org/).

## Results

### Baseline characteristics of the population

**Fig. 1:**
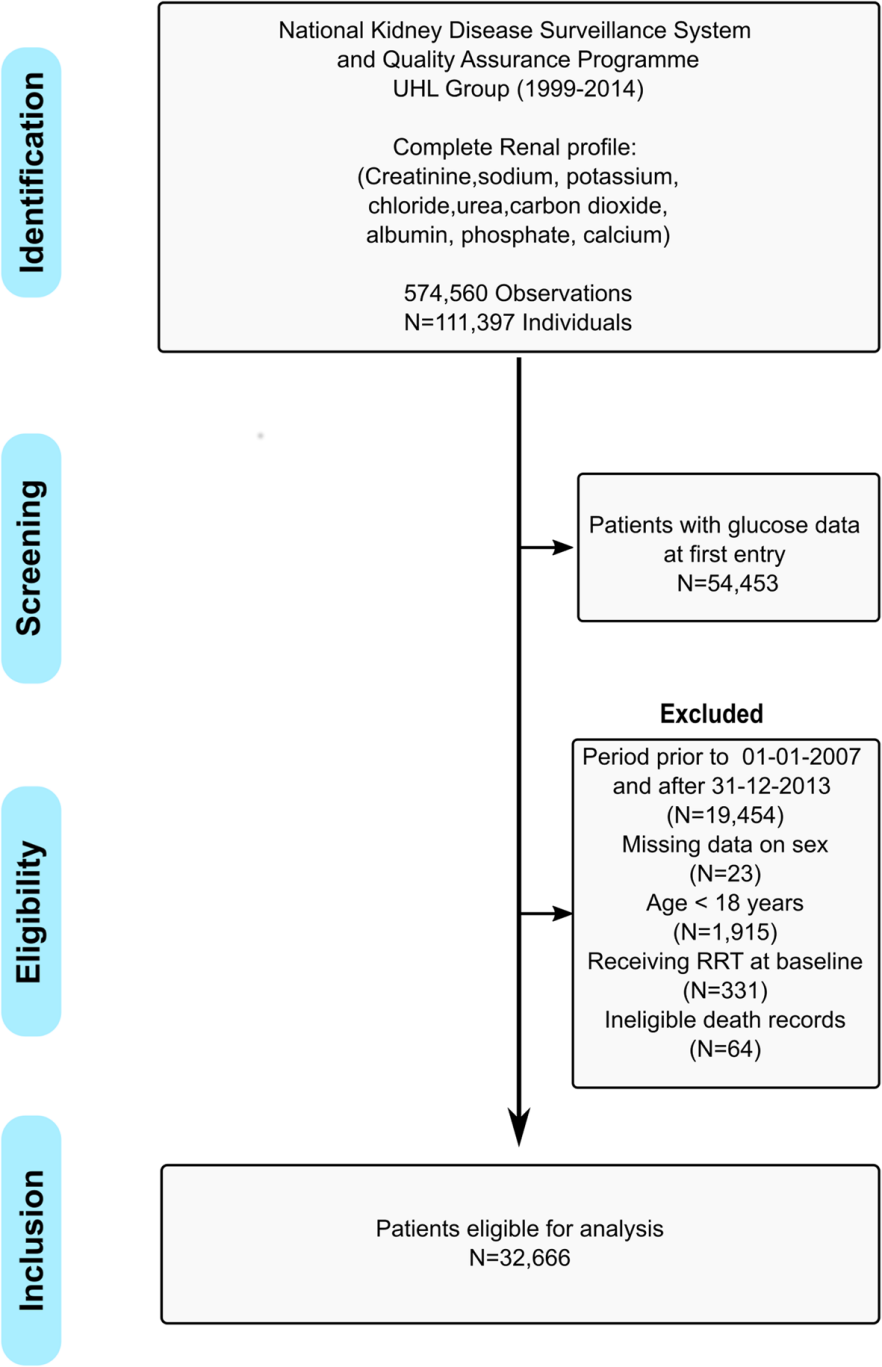
Strobe diagram for the cohort study from 2007–2013. The final dataset captured information on demographic characteristics, clincal settings, measured laboratory parameters and mortality

The construction of the final study cohort from the Midwest region of Ireland is shown in Fig. 1 according to the STROBE criteria. The distributions of demographic characteristics and laboratory measures by baseline serum sodium concentration are provided in Table 1. Of the 32,666 patients, 47% were male and mean (SD) age was 56.9 (18) years. The prevalence of hypo- and hypernatraemia was 7.7% and 0.8% respectively at baseline. During follow-up, 14.2% sustained at least one episode of hyponatremia, while 2.3% experienced at least one episode of hypernatremia. Compared to patients with normal serum sodium concentrations, patients classified as having hyponatraemia at baseline were on average older, had poorer kidney function, and lower haemoglobin and serum albumin levels. These patients also had higher levels of inflammatory markers (white cell count, lymphocyte count, neutrophil count) and higher blood glucose levels. Patients classified as having hypernatraemia had similar profiles to those of hyponatraemic patients with lower levels of kidney function, lower haemoglobin values and higher concentration of inflammatory markers. The majority of blood tests documenting serum sodium levels were based in primary care, followed by inpatient setting with the remainder spilt between outpatient and emergency departments. The distribution of baseline characteristics by CKD status and clinical setting are available in Tables S 1 and S 2.

**Table 1:** Baseline characteristics of patients by serum Sodium concentration (mmol/L) in the Irish Health System

		**Serum Sodium Category**			***P*** **-value**
**Variable**	**N**	** < 135 mmol/L**	**135–145 mmol/L**	** > 145 mmol/L**	
Observations n (%)	32,666	2503 (7.7)	29,908 (91.6)	255 (0.8)	
Mean Age at baseline (SD)	32,666	65.9 (16.8)	56.1 (17.9)	63.3 (18.3)	< 0.001
**Sex**					
Women	17,367	48.8	53.6	44.7	
Men	15,299	51.2	46.4	55.3	< 0.001
**Clinical setting at baseline **^**a**^					
Emergency Department	4,835	30.1	13.5	16.9	
General Practice	15,293	19.7	49.4	21.2	
Inpatient	7,543	37.7	21.6	56.1	
Outpatient	4,965	12.5	15.5	5.9	< 0.001
**Markers of renal function**					
Urea (mmol/L) (Median IQR)	32,666	5.3 (3.8–8.2)	4.9 (3.8–6.2)	7.9 (5.2–14.1)	< 0.001
Serum creatinine (µmol/L) (Median IQR)	32,666	81.0 (64.0–108.0)	77.0 (65.0–91.0)	97.0 (78.0–140.0)	< 0.001
Baseline eGFR ^b^ (ml/min/1.73m^2^) (Median IQR)	32,666	76.5 (50.9–93.6)	86.4 (68.5–101.2)	63.1 (36.3–86.7)	< 0.001
**eGFR Category (ml/min/1.73m**^**2**^**) **^**b**^					
eGFR > = 90	13,882	30.1	43.7	20.8	
eGFR 60–89	12,867	36.8	39.7	33.7	
eGFR 30–59	4,755	21.9	13.8	29.8	
eGFR 15–29	814	7	2	11.8	
eGFR < 15	348	4.4	0.8	3.9	< 0.001
**Inflammatory Markers**					
Haemoglobin (g/dl) (Mean SD)	26,561	12.4 (2.1)	13.5 (1.8)	12.4 (2.5)	< 0.001
White blood count (× 10^9^/L) (Median IQR)	26,561	9.2 (6.7–12.7)	7.2 (5.8–9.4)	10.5 (7.2–15.0)	< 0.001
Lymphocyte count (× 10^9^/L) (Median IQR)	26,561	1.2 (0.8–1.7)	1.7 (1.3–2.2)	1.3 (0.8–1.9)	< 0.001
Neutrophil count (× 10^9^/L) (Median IQR)	26,561	6.8 (4.4–10.4)	4.4 (3.3–6.3)	8.4 (4.7–12.3)	< 0.001
**Nutritional and Metabolic Markers**					
Serum Albumin (g/L) (Mean SD)	32,666	32.6 (7.3)	37.7 (5.5)	29.7 (9.5)	< 0.001
Serum Calcium (mmol/L) (Mean SD)	32,666	2.2(0.2)	2.3 (0.1)	2.2 (0.3)	< 0.001
Serum Phosphorus (mmol/L) (Mean SD)	32,666	1.2(0.4)	1.2 (0.3)	1.2 (0.5)	< 0.001
Serum Potassium (mmol/L) (Mean SD)	32,666	4.3(0.8)	4.4 (0.5)	4.1 (0.8)	< 0.001
**Lipid related Markers**					
Total Cholesterol (mmol/L) (Mean SD)	17,188	4.7 (1.3)	5.1 (1.1)	4.9 (1.2)	< 0.001
Triglycerides (mmol/L) (Mean SD)	14,302	1.3 (1.1)	1.4 (0.9)	1.6 (1.2)	< 0.001
**Glycaemic markers**					
Glucose (mmol/L) (Median IQR)	32,666	6.6 (5.4–10.1)	5.2 (4.8–6.1)	6.2 (5.2–8.0)	< 0.001
**Markers of Liver function**					
Alanine Alkaline phosphatase (IU/L) (Median IQR)	31,599	79.0 (61.0–105.0)	68.0 (55.0–84.0)	75.0 (57.2–97.0)	< 0.001
Alanine transaminase (IU/L) (Median IQR)	31,131	23.0 (17.0–35.0)	23.0 (17.0–32.0)	24.0 (18.0–42.0)	0.018
Gamma-glutamyltransferase (IU/L) (Median IQR)	31,196	34.0 (21.0–67.0)	24.0 (17.0–39.0)	31.0 (21.0–70.0)	< 0.001
Total bilirubin (µmol/L) (Median IQR)	30,867	13.0 (10.0–19.0)	12.0 (9.0–16.0)	14.0 (10.0–21.0)	< 0.001

^a^Clinical setting at baseline refers to the clinical location of patient where the laboratory test was taken

^b^eGFR: Estimated glomerular filtration rate (ml/min per 1.73 m^2^) was based on the Chronic Kidney Disease Collaborative (CKD-EPI) [18].

### Serum sodium and all-cause mortality

Over a median follow up time of 5.45 (3.90–6.33) years 5,114 patients (15.7%) died. Crude mortality rates were highest for hypernatraemia (131 per 1,000 person years) and lowest for normonatraemia (30 per 1,000 person years). Kaplan–Meier (S 1 Fig.) curves and Cox proportional hazards analyses revealed significantly higher all-cause mortality for patients classified as hyponatraemic and hypernatraemic at baseline compared to patients with serum sodium concentrations in the normal range. After adjusting for confounding variables, hyponatraemia [hazard ratio (95% CI): 1.31 (1.21–1.43)] and hypernatraemia [HR (95% CI): 1.45 (1.17–1.79)] from baseline serum sodium levels were associated with all-cause mortality. The multivariable time-dependent models further confirmed significantly higher mortality risks for patients classified as hyponatraemic [(HR (95% CI):1.80 (1.67–1.94)] and hypernatraemic [(HR (95%CI): 2.43 (2.06–2.88)] and these estimates were of greater magnitude than those estimated by single baseline sodium values (Table 2, Table S 3).

**Table 2:** Associations between Serum sodium, all-cause and cause-specific in time-varying Cox Models

	** < 135 mmol/L**	**135–145 mmol/L** **Reference Group**	** > 145 mmol/L**
Hazard Ratio for all-cause mortality (95% CI)	1.80 (1.67–1.94)	1.00	2.43 (2.06–2.88)
**Cause-Specific Hazard Model (95% CI)**			
Cardiovascular deaths	1.38 (1.18–1.61)	1.00	2.16 (1.58–2.96)
Malignancy-related deaths	2.49 (2.23–2.78)	1.00	1.05 (0.66–1.66)
Non-cardiovascular/non-malignancy-related deaths	1.36 (1.17–1.58)	1.00	3.60 (2.87–4.52)

Final model was adjusted for demographic factors (baseline age, sex), clinical indicators (haemoglobin, estimated glomerular filtration rate, serum albumin, serum potassium, serum calcium, white blood cell count, alanine aminotransferase, alkaline phosphatase, and clinical setting at baseline

**Fig. 2:**
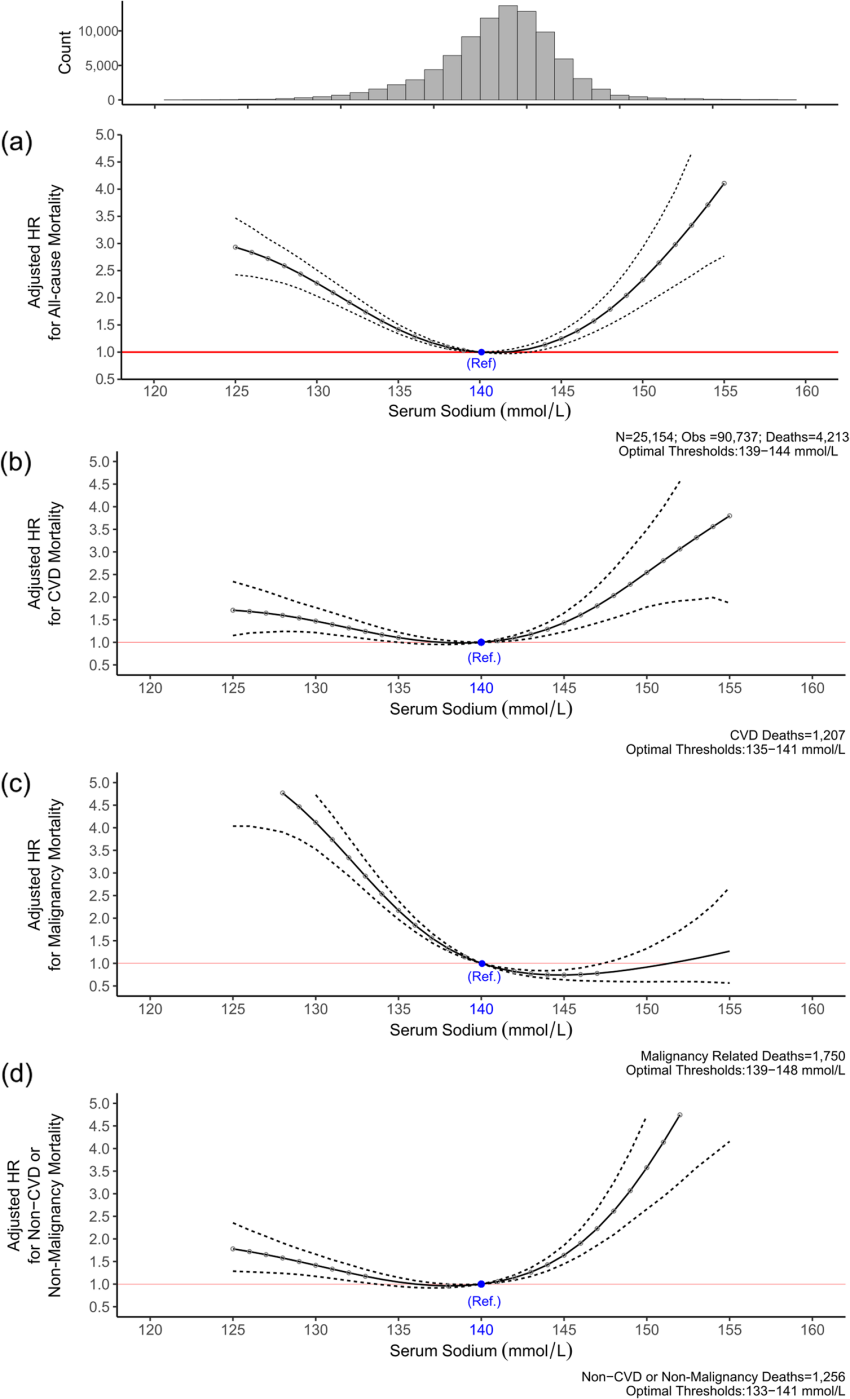
Adjusted hazard ratios for for all-cause and cause specific mortality by serum sodium. Solid line (—) denotes Hazard ratio and dash line (-—-) denotes 95% confidence intervals (CIs). Filled circles denote statistical significance (P < 0.05) compared with the reference (blue circle). Estimates are based on unadjusted and adjusted penalised spline models for the association between time dependent serum sodium and all-cause mortality. Hazard ratios were adjusted for age, sex, estimated glomerular filtration rate, serum albumin, haemoglobin, serum potassium, serum calcium, white blood cell count, alanine aminotransferase, alkaline phosphatase, and clinical setting at baseline

When serum sodium was modelled as a continuous time-dependent repeated measure using penalised spline models, a non-linear U-shaped association was observed with mortality (p-value for test of linearity < 0.001) (Fig. 2). This U‐shaped association persisted after adjusting for confounding. The optimal threshold values for serum sodium associated with the lowest mortality were determined to lie between 139 mmol/L [HR 1.03 (1.01–1.05)] and 144 mmol/L [HR 1.14 (1.05–1.24)]. Serum sodium values beyond this range were associated with increased mortality risk compared to a referent value of 140 mmol/L.

### Serum sodium and Cause-specific mortality

Table S 4 shows the main causes of death by sodium category at baseline. Compared to normonatraemia, hyponatremia was significantly associated with increased risk of death from cardiovascular causes [HR 1.38 (1.18–1.61)], malignancy [HR: 2.49 (2.23–2.78)] and non-cardiovascular/non-malignant causes [1.36 (1.17–1.58)] (Table 2) in the multivariable models, while hypernatremia was significantly associated with increased risk of both cardiovascular [HR: 2.16 (1.58–2.96)] and non-cardiovascular/ non-malignancy mortality respectively [HR: 3.60 (2.87–4.52)].

**Fig. 3:**
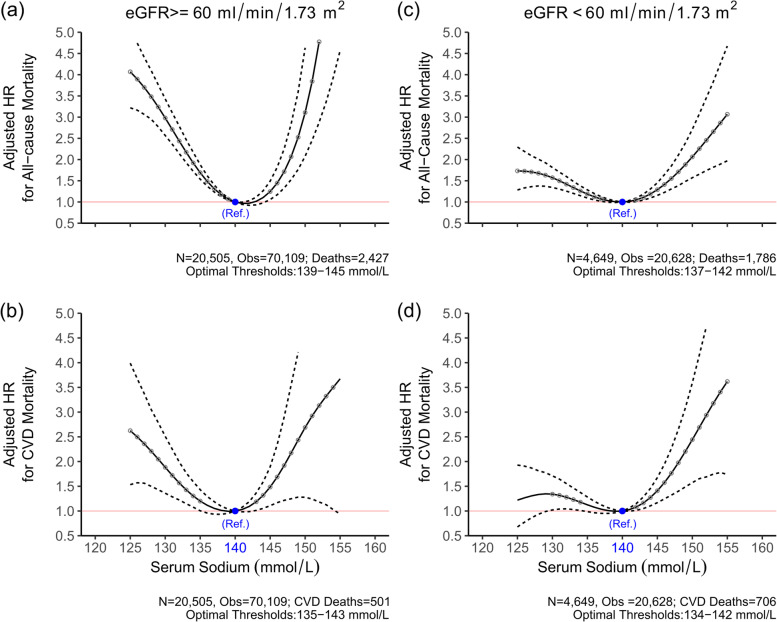
Adjusted hazard ratios for all-cause and cardiovascular mortality by serum sodium stratified by eGFR category. Solid line (—) denotes Hazard ratio and dash line (-—-) denotes 95% confidence intervals (CIs). Filled circles denote statistical significance (P < 0.05) compared with the reference (blue circle). Estimates are based on unadjusted and adjusted penalised spline models for the association between time dependent serum sodium and all-cause mortality. Hazard ratios were adjusted for age, sex, serum albumin, haemoglobin, serum potassium, serum calcium, white blood cell count, alanine aminotransferase, alkaline phosphatase, and clinical setting at baseline

In time-dependent models, serum sodium followed a U-shaped pattern with cardiovascular mortality with values of 135–141 mmol/L associated with optimal survival (Fig. 3). The pattern was similar for non-cardiovascular/malignancy deaths with an optimal range of 133–141 mmol/L (referent value 140 mmol/L). However, for deaths due to malignancy, the shape of the association changed with a sharp rise in mortality risk for patients with low serum sodium values but not for those with higher concentrations.

### Effect modifiers

Several significant interactions between serum sodium and mortality were found for kidney function, age and designated clinical setting. For hyponatremia, interactions indicated that while significant at most levels, the increased mortality hazard associated with hyponatremia was greater for younger patients (P < 0.001) than older patients, for patients with higher eGFR levels (P < 0.001) and for patients attending general practice (P < 0.001) than other clinical settings (Table 3). The effect modifiers for hypernatremia and mortality were similar to those of hyponatreamia, although age-adjusted rates of mortality were greatest for inpatients and those in the emergency depatment (Table S 8). Relative mortality risks associated with hypernatraemia were greatest for patients in the outpatient setting (HR 9.84, 95% CI 4.88–18.62) comapred to other clinical settings at baseline.

**Table 3:** Interactions between Sodium category, eGFR, age and clinical setting at baseline on all-cause mortality

	**Serum Sodium Category**			***P***-interaction
**Variable**	** < 135 mmol/L** **HR (95%CI)**	**135–145 mmol/L** **HR (95%CI)**	** > 145 mmol/L** **HR (95%CI)**	
**Model with eGFR interaction**				< 0.001
eGFR > = 60 ml/min/1.72m^2^	2.28 (2.04–2.51)	1.00 (Ref.)	3.28 (2.19–4.72)	
eGFR < 60 ml/min/1.72m^2^	1.31 (1.13–1.49)	1.00 (Ref.)	2.02 (1.44–2.68)	
**Model with age interaction**				< 0.001
Age 40	3.33 (2.72–4.02)	1.00 (Ref.)	6.96 (3.98–10.76)	
Age 50	2.67 (2.31–3.07)	1.00 (Ref.)	4.85 (3.16–6.75)	
Age 60	2.14 (1.93–2.35)	1.00 (Ref.)	3.40 (2.55–4.34)	
Age 70	1.72 (1.58–1.85)	1.00 (Ref.)	2.40 (1.91–2.96)	
Age 80	1.38 (1.25–1.50)	1.00 (Ref.)	1.70 (1.27–2.23)	
**Model with Clinical setting interaction**				< 0.001
Emergency Department	1.19 (0.99–1.41)	1.00 (Ref.)	3.09 (1.89–4.88)	
General Practice	2.70 (2.15–3.36)	1.00 (Ref.)	3.13 (1.20–5.46)	
Inpatient	1.69 (1.50–1.89)	1.00 (Ref.)	1.61 (1.15–2.17)	
Outpatient	1.90 (1.54–2.31)	1.00 (Ref.)	9.84 (4.88–18.62)	

Final Model was adjusted for baseline age, sex, eGFR, albumin, haemoglobin, potassium, calcium, white blood cell count, alanine aminotransferase, alkaline phosphatase and clinical setting at baseline

**Fig. 4:**
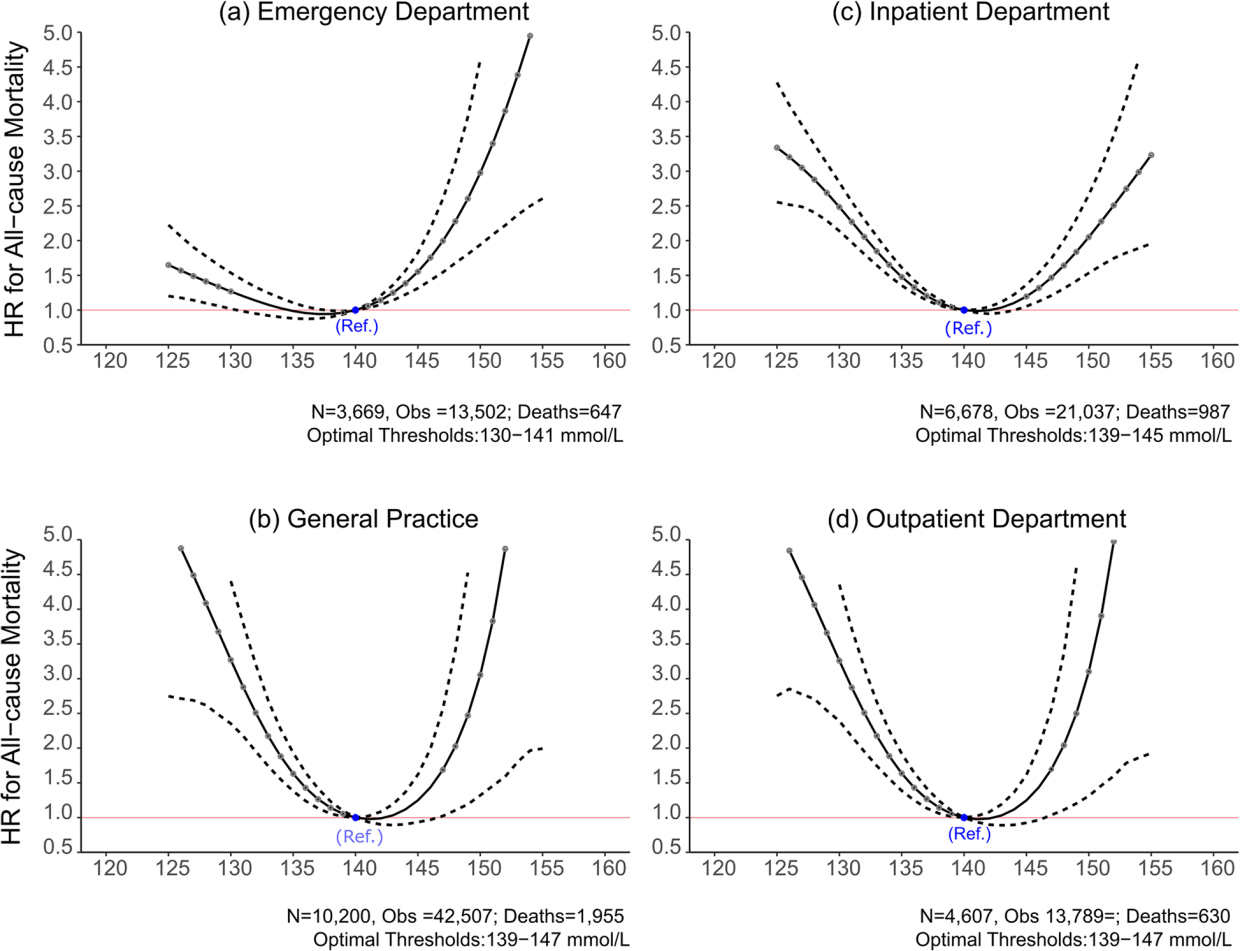
Adjusted hazard ratios for all-cause mortalityby serum sodium stratified by clinincal setting at baseline Solid line (—) denotes Hazard ratio and dash line (-—-) denotes 95% confidence intervals (CIs). Filled circles denote statistical significance (P < 0.05) compared with the reference (blue circle). Estimates are based on adjusted penalised spline models for the association between time dependent serum sodium and all-cause mortality. Hazard ratios were adjusted for age, sex, estimated glomerular filtration rate, serum albumin, haemoglobin, serum potassium, serum calcium, white blood cell count, alanine aminotransferase, alkaline phosphatase

Among patients with hyponatraemia, mortality risks increased in a graded fashion from the oldest to the youngest age group [HR 1.38 (1.25–1.50) for age 80, to HR 3.33 (2.72–4.02) for age 40]. As expected, the absolute risk of mortality was higher in individuals with CKD than those without CKD based on an eGFR threshold of 60 ml/min/1.73m^2^ as shown in Table S 7. A significant interaction (p < 0.001) revealed that the pattern of risk varied by CKD status, with a predominantly U-shaped pattern for patients with GFR ≥ 60 ml/min/1.73m^2^ and a more flattened curve for those with GFR < 60 ml/min/1.73m^2^ (Fig. 4,Fig S 2). Furthermore, the mortality thresholds also differed by CKD status as shown in the stratified results. For those without CKD the optimal serum sodium range was 139 to 145 mmol/L for all–cause mortality and 135 to 143 mmol/L for CVD related mortality. For those with CKD, the risk thresholds shifted ranging from 137 to 142 mmol/L for all–cause mortality and from 134 to 142 mmol/L for CVD mortality.

The sodium-mortality relationship was also influenced by the clinical setting at baseline. The age-adjusted mortality rates were greatest for patients classified as inpatients and in the emergency department and were lowest for patients classified as outpatient or in general practice resepctively (Table S 8). Among patients with hyponatraemia, the relative risk of mortality was greatest for general practice HR 2.70 (2.15–3.36), and lowest for patients in the emergency department HR 1.19 (0.99–1.41). Similarly, among hypernatraemia patients, mortality risks were significantly higher for those who attended the outpatient department HR 9.84 (4.88–18.62) than other clinical settings especially inpatient setting HR 1.61 (1.15–2.17) (Table 3). Serum sodium concentrations associated with the lowest all-cause mortality ranged from 139 to 147 mmol/L in general practice and in outpatient settings, and this was similar for inpatients (139 to 145 mmol/L). Serum sodium concentrations beyond 130–141 mmol/L range were associated with increased mortality risk in the emergency department (Fig. 4).

## Discussion

In this large population-based study across a variety of clinical settings, we report significant associations of both higher and lower levels of serum sodium with increased all-cause mortality. The pattern of association was primarily U-shaped and persisted following adjustment that suggesting dysnatraemic states are independent risk factors for mortality. Hyponatremia was associated with higher risks for all-cause and cause specific mortality, while hypernatremia correlated with higher all-cause, cardiovascular and non-cardiovascular/non-malignancy-related deaths suggesting potential mechanistic pathways for these causes of death. Importantly, we discovered that mortality risks varied across specific demographic and clinical subgroups. We noted that relative mortality risks were magnified in younger patients and in patients with better levels of kidney function although the absolute risk of mortality was lowest in these groups. Furthermore, we determined that the clinical setting at baseline also influenced the magnitude of associated risk for both hyper- and hyponatraemia, suggesting that these areas should be considered a target for regular surveillance and timely intervention.

The frequency with which dysnatraemia occurs in clinical practice and potential consequences makes it a prime target for regular surveillance and early intervention [1, 2, 11–13]. A key focus of the present study was to explore patterns of mortality risk across the spectrum of serum sodium concentrations to identify specific threshold values and explore whether these patterns were influenced by patient factors and primary clinical settings. In this large cohort of patients across multiple clinical settings that included both ambulatory and hospitalised patients, we found that hyponatremia and hypernatremia were independently associated with elevated mortality risk. Beyond the standard definitions for hypo-and hypernatremia, we observed a U-shaped association between serum sodium and mortality with the optimal range for survival falling between 139 mmol/L and 144 mmol/L. These threshold values are in close agreement but not directly concordant with the standard normal range for serum sodium values in clinical practice (normal range 135–145 mmol/L). Our analysis confirmed increased mortality risk for patients with low-normal serum sodium values of 135–139 mmol/L, values that are typically considered in the healthy range and extend the findings of other investigators [12, 13]. These observations would suggest that consideration be given to adjusting optimal threshold values for serum sodium concentrations in clinical practice in order to account for mortality impact.

A unique feature of the present study was the identification of key effect modifiers of the relationship between sodium concentration and mortality. The age interaction was quite striking and revealed that the younger the age, the greater the impact of both hyponatraemia and hyponatraemia on mortality. For hyponatraemia, mortality risks increased in a graded fashion from the oldest to the youngest age group [HR 1.38 (1.25–1.50) for age 80, to HR 3.33 (2.72–4.02) for age 40], mortality risks were more than 2 times greater for patients age 40 years than for patients age 80 years, all else being equal. For hypernatraemia, the magnitude of difference between the youngest and oldest age groups was 4 times greater. The inference is that an abnormally low or high serum sodium is more deleterious in younger patients than in older patients. Whether this reflects the severity of the underlying illness in younger patients or the presence of other competing mortality risk factors in older patients deserves further study [7].

The most striking interaction noted in our analysis was the effect modification by kidney function. The impact of dysnatraemia on mortality was of greater magnitude in those without CKD than with CKD, although the absolute mortality for CKD patients was greater than that for non CKD (S 2 Fig.). A graded risk pattern was observed with increasing GFR that was not explained with adjustment for several indicators of health status. The spline analyses confirmed a more flattened curve for patients with GFR < 60 ml/min/1.72m^2^ and a shift in risk thresholds compared to those with higher GFR values. Serum sodium is regulated through water intake, via thirst sensation, and water excretion, via the actions of anti-diuretic hormone (ADH) on the collecting ducts of the nephron [1, 9, 13]. Disruption of these homeostatic mechanisms may lead to abnormal dilution or concentration of the extracellular fluid resulting in hypo- or hypernatraemia. Kovesdy et al. advanced the hypothesis that the chronic nature of the abnormalities affecting water metabolism in CKD may allow the body to adapt to these consequences and thus mitigate the impact on adverse outcomes [13, 19]. This adaptive mechanism may explain why the U-shaped association between serum sodium and mortality is flattened in patients with lower levels of kidney function, and why there is a very clear shift in sodium thresholds for mortality (optimal range: 137–142 mmol/L). Moreover, the greater mortality impact of hypernatremia may arise from its higher incidence among patients with CKD as the concentrating capacity of the kidney becomes impaired more readily than the diluting capacity. These findings should prompt consideration for disease-specific thresholds for sodium concentrations as opposed to a single range of sodium concentrations for all patients. Optimal thresholds for serum sodium based on real-life survival data may provide a more meaningful target for serum sodium values in clinical practice rather than our current practice of using values derived from cross-sectional population surveys.

There is controversy as to whether dysnatraemia itself directly leads to death or whether it is merely a risk marker of underlying serious disease [7]. On one hand, there is evidence to support a direct causal relationship as severe dysnatraemia may precipitate dangerous sequelae including impaired brain function, reduced cardiac contractility, and increased insulin resistance, abnormalities in neuromuscular function and interstitial oedema [20–23]. A recent study by Lombardi et al. further implicates the magnitude of sodium fluctuations as an independent contributor to mortality beyond baseline sodium concentrations [24], these pathophysiological changes may be directly linked to mortality [12, 13, 25, 26]. Our analysis lends support to the causal hypothesis, as serum sodium concentrations were significantly associated with elevated mortality despite adjustment for several acute illness indicators, and in a variety of clinical settings [13]. On the other hand, it is equally very likely that fluctuations in serum sodium represent a surrogate marker for heath and that the extent of dysnatraemia may reflect the extent and severity of underlying burden of disease [3, 6, 13, 26]. Our results suggest that the pattern of risk varies by patient subgroups and across clinical settings suggesting that threshold of risk is influenced by factors other than serum sodium concentrations. A review of the medical records of 53 patient who died after developing severe hyponatraemia by Chawla et al. found that the nature of underlying illness rather than the severity of hyponatremia best explained mortality [7].

The findings of the present study have a number of important clinical implications. First, this study estimated the range of serum sodium concentrations that correlated with optimal survival (139–144 mmol/L) and found that it differed from the standard laboratory range for serum sodium. Second, we identified for the first time a number of important effect modifiers of the sodium-mortality relationship including age, level of kidney function and clinical setting at baseline, and challenge the use of standard cut-off thresholds for all patients. Third, we found that sodium disorders have effects on multiple systems as we report that low sodium levels were associated with increased mortality from cardiovascular causes, malignancy and non-cardiovascular/non-malignant deaths. Whereas, abnormally high sodium concentrations were associated with cardiovascular causes and non-cardiovascular/non-malignant deaths suggesting that these may be the mechanisms through which risk is conferred. Finally, we further extend the observations of previous studies confirming that dysnatraemia is an independent predictor of mortality and should be included in mortality risk models.

This study has several strengths. First, while previous studies have largely focused on very selective in-patient and ICU cohorts [27], our study is one of the first to examine the mortality risk associations across a variety of clinical settings thereby providing a real world insight into burden and impact. Second, the inclusion and adjustment for a large selection of clinical heath indicators and the use of time-dependent repeated measures analyses minimised the impact of confounding imparted by liver disease, impaired kidney function, anaemia, malnutrition and inflammation. Third, the availability of data on serum creatinine, GFR and clinical setting at baseline allowed us to fully explore effect modifiers of the sodium-mortality relationship. Finally, linkage to data on cause of death from the Central Statistics Office allowed us a unique opportunity to explore reasons for mortality that may be causally linked to a wide spectrum of serum sodium concentrations.

This study also has some inherent limitations. Our study was observational, and we recognise the potential for residual confounding. Although information on many clinically relevant variables was collected and we performed careful statistical adjustments in the analyses to account for differences between exposure groups, unmeasured confounders may still be present. It is conceivable that the relative importance of hyponatremia decreases with lower eGFR as the underlying absolute risk of mortality is larger and patients are at increased of risk of death due to other causes. We recognise that hyponatremia is also less common in individuals with normal renal function. Thus, the increased risk of mortality in this group may be more a proxy of severe acute illnesses (detection bias). Similarly, the association of hyponatremia with mortality observed among younger patients may be a proxy of severe acute illness not captured or reflected in our dataset. A number of studies have documented an association between hyponatremia and increased mortality from diseases involving fluid overload and diuretic use in heart failure patients and liver cirrhosis with few studies exploring the association in CKD populations [28, 29]. Due to a lack of medication data we are unable to test whether long term use of these medications or resistance to them account for the shift in the mortality risk threshold among CKD patients. Given the multifactorial nature of dysnatraemia and varying presentation, it was not possible to balance patient characteristics by number and age profiles. Our dataset did not capture information on urine osmolality, malignancy, heart failure, comorbid conditions and medications which may have influenced the nature and magnitude of observed associations. As such, these findings may not be specific to any of these particular subgroups which limits the degree of risk stratification. For malignancy-related deaths we were unable to discern whether these deaths were due to pre-existing malignancy as hyponatremia may arise as a symptom of malignancy or due to treatment such as chemotherapy or immunotherapies [30], such treatment effects could partly explain the reduced risk for hypernatremia for this outcome. The rate of correction of dysnatraemia is linked to mortality thus; information on this may have affected the results.

## Conclusion

In summary, our study supports evidence of a U-shaped association between serum sodium and mortality with a range of values associated with the greatest survival falling between 139 mmol/L and 144 mmol/L. In this study, demographic factors or markers of acute and chronic illness did not explain the observed increase in mortality. The mortality risks from hyponatraemia were in part due to excess deaths from cardiovascular causes, malignancy, and non-cardiovascular/non-malignant conditions, whereas deaths associated with hypernatremia were driven by cardiovascular, non-cardiovascular, and non-malignancy-related deaths. These relationships were influenced significantly by age, level of kidney function and the clinical setting at baseline. The findings reported here suggest that a range of values for serum sodium supporting optimal health be considered in the context of patient survival and that prognostic models of risk include serum sodium as an independent predictor.

## Supplementary Information

**Additional file 1:** **S1 Fig.** Unadjusted Kaplan-Meier survival curves of patients with hyponatremia and hypernatremia at baseline.

**Additional file 2:** **S2 Fig.** Adjusted relative mortality risk associated with Na+ levels by eGFR category. Red solid line (—) denotes Hazard ratio and dash line (- - -) denotes 95% confidence intervals (CIs) for eGFR ≥ 60 ml/min/1.72m^2^. Blue solid line (—) denotes Hazard ratio and the ribbon denotes 95% confidence intervals (CIs) for eGFR < 60 ml/min/1.72m^2^. Hazard ratios were adjusted for age, sex,  serum albumin, haemoglobin, serum potassium, serum calcium, white blood cell count, alanine aminotransferase, alkaline phosphatase, and clinical location at baseline and an interaction term between serum sodium and eGFR was included.

**Additional file 3: Supplementary Table 1.** Baseline Demographic and Clinical Characteristics of Study Population by CKD Status*.*

**Additional file 4: Supplementary Table 2.** Baseline Demographic and Clinical Characteristics of Study Population by Clinical Setting at Baseline*.*

**Additional file 5: Supplementary Table 3.** Relationship of Serum Sodium with All-Cause Mortality in the Health System.

**Additional file 6: Supplementary Table 4.** Leading causes of death by baseline levels of Serum Sodium concentration.

**Additional file 7:Supplementary Table 5.** Age-adjusted mortality rates per 1,000 years of follow up by baseline serum sodium level*. **Supplementary Table 6.** Age-adjusted All-cause mortality rates per 1,000 years of follow up by baseline serum sodium level for specific age values*.**Supplementary Table 7.** Age adjusted mortality rates per 1,000 years of follow up by baseline serum sodium level* stratified by CKD subgroup.**Supplementary Table 8.** Age adjusted mortality rates per 1,000 years of follow up by baseline serum sodium level* stratified by clinical setting at baseline.

## Data Availability

The datasets used and/or analysed during the current study are available from the corresponding author on reasonable request.
