# Supplementary figures and images for: Impact of serum sodium concentrations, and effect modifiers on mortality in the Irish Health System
Source: BMC Nephrol. 2023 Jul 6;24:203. doi: 10.1186/s12882-023-03251-w (PMC10324141; doi:10.1186/s12882-023-03251-w)

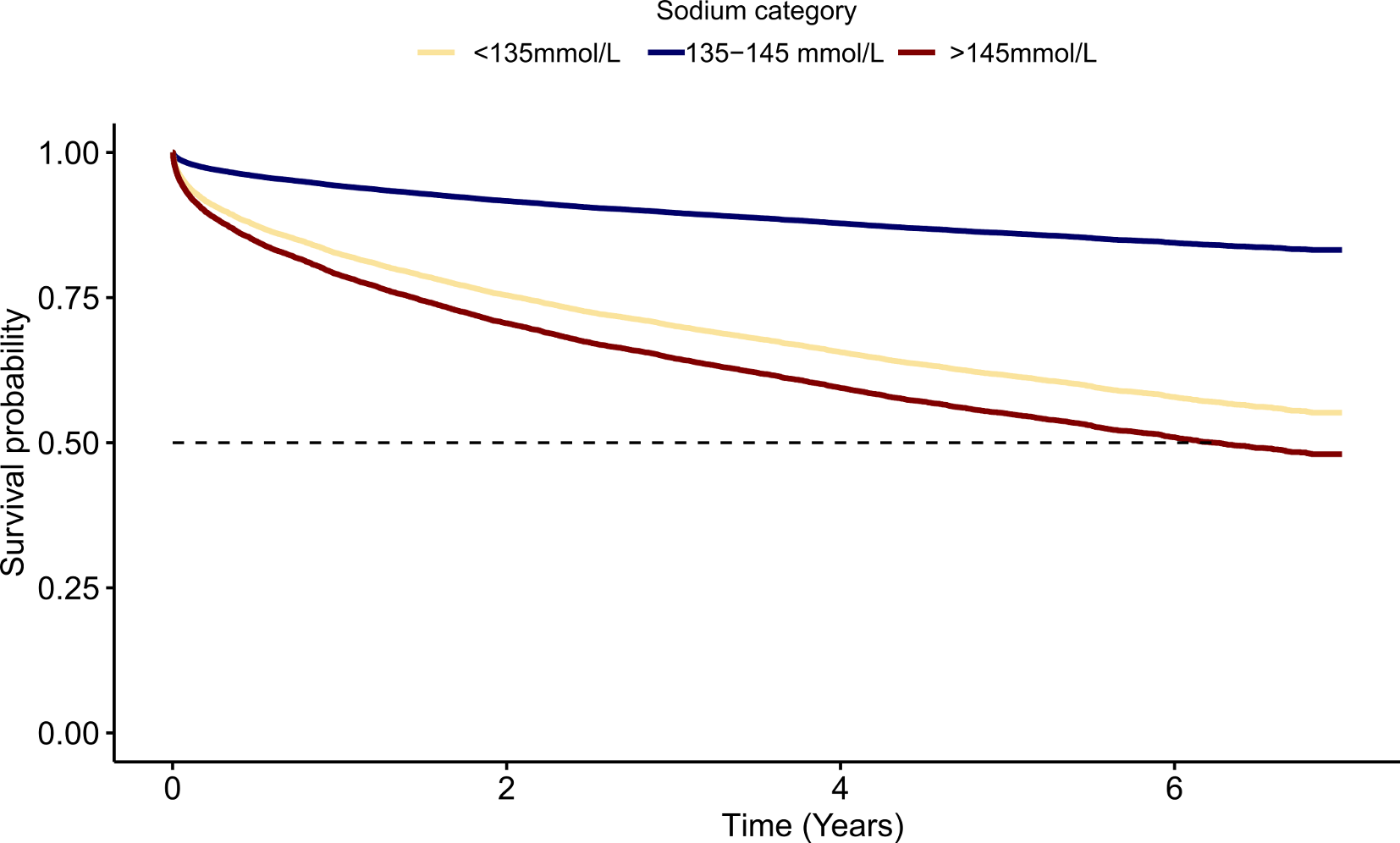

Supplement: Supplementary file 1 — Additional file 1: S1 Fig. Unadjusted Kaplan-Meier survival curves of patients with hyponatremia and hypernatremia at baseline. [file 12882_2023_3251_MOESM1_ESM.tiff]

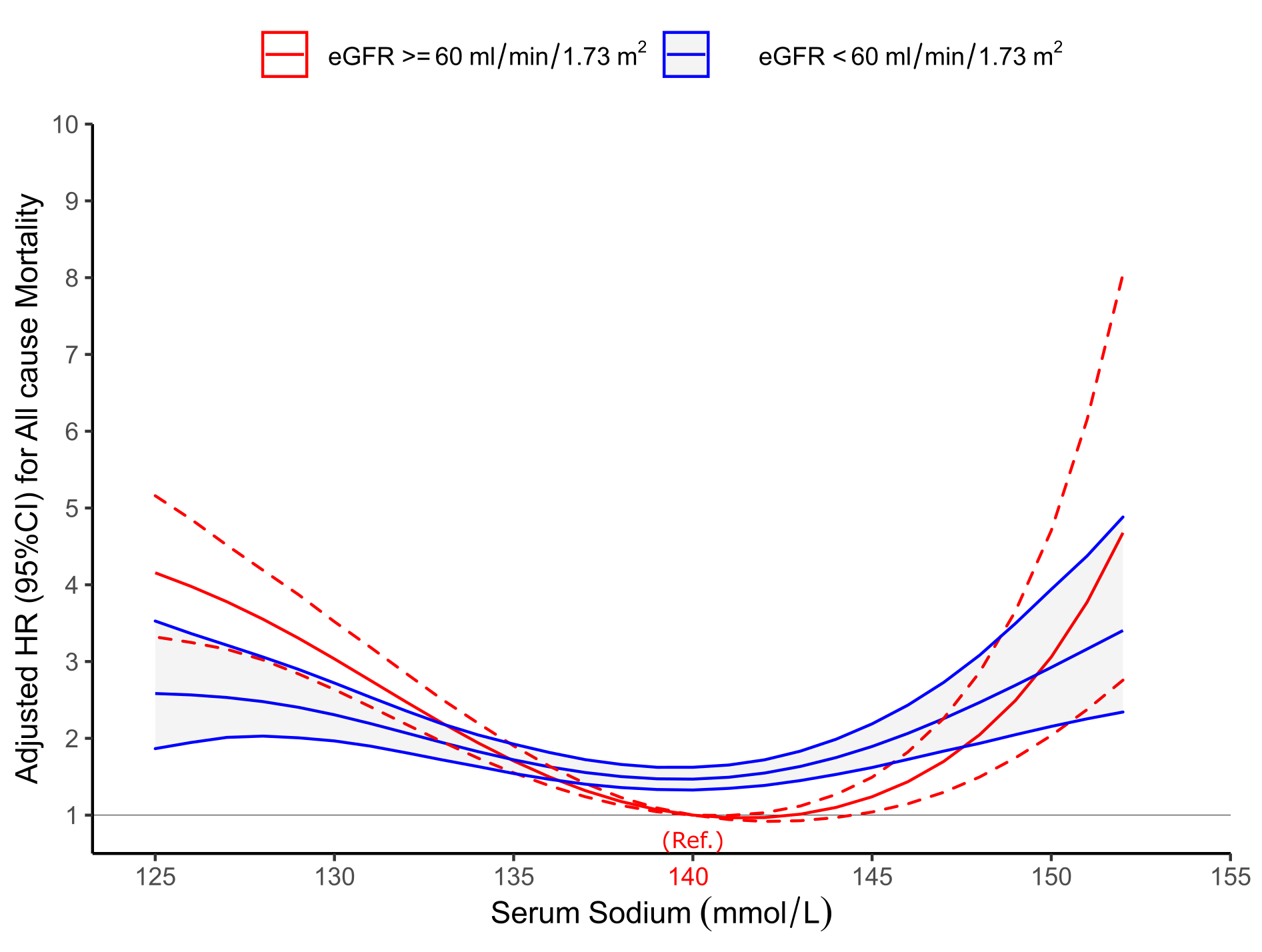

Supplement: Supplementary file 2 — Additional file 2: S2 Fig. Adjusted relative mortality risk associated with Na+ levels by eGFR category. Red solid line (—) denotes Hazard ratio and dash line (- - -) denotes 95% confidence intervals (CIs) for eGFR ≥ 60 ml/min/1.72m2. Blue solid line (—) denotes Hazard ratio and the ribbon denotes 95% confidence intervals (CIs) for eGFR < 60 ml/min/1.72m2. Hazard ratios were adjusted for age, sex, serum albumin, haemoglobin, serum potassium, serum calcium, white blood cell count, alanine aminotransferase, alkaline phosphatase, and clinical location at baseline and an interaction term between serum sodium and eGFR was included. [file 12882_2023_3251_MOESM2_ESM.tiff]
