# Supplementary material for: Impact of serum sodium concentrations, and effect modifiers on mortality in the Irish Health System
Source: BMC Nephrol. 2023 Jul 6;24:203. doi: 10.1186/s12882-023-03251-w (PMC10324141; doi:10.1186/s12882-023-03251-w)
Supplement: Supplementary file 3 — Additional file 3: Supplementary Table 1. Baseline Demographic and Clinical Characteristics of Study Population by CKD Status. [file 12882_2023_3251_MOESM3_ESM.docx]

**Supplementary Table 1. Baseline Demographic and Clinical Characteristics of Study Population by CKD Status**

| **Variable** | **N** | **GFR >=60**  **ml/min/1.73m^2^** | **GFR <60**  **ml/min/1.73m^2^** | **P-value** |
| --- | --- | --- | --- | --- |
| Observations n (%) | 32,666 | 26,749 (81.9) | 5,917 (18.1) |  |
| Mean Age at baseline (SD) | 32,666 | 53.1(16.9) | 74.0 (12.1) | <0.001 |
| **Sex** |  |  |  |  |
| Women | 17,367 | 52.8 | 54.9 |  |
| Men | 15,299 | 47.2 | 45.1 | 0.004 |
| **Clinical Setting at Baseline ^a^** |  |  |  |  |
| General Practice | 15,293 | 51.2 | 27.2 |  |
| Emergency Department | 4,835 | 13.1 | 22.7 |  |
| Inpatient | 7,543 | 20.7 | 34.1 |  |
| Outpatient | 4,965 | 15.1 | 15.9 | <0.001 |
| **Markers of renal function** |  |  |  |  |
| Urea (mmol/L) (Median IQR) | 32,666 | 4.6 (3.6-5.6) | 8.3 (6.3-11.7) | <0.001 |
| Serum creatinine (µmol/L) (Median IQR) | 32,666 | 73.0 (63.0-84.0) | 117.0 (99.0-151.0) | <0.001 |
| Baseline eGFR ^b^ (ml/min/1.73m^2^) (Median IQR) | 32,666 | 91.0 (78.1-103.8) | 46.2 (33.6-54.1) | <0.001 |
| **Inflammatory Markers** | | | | |
| Haemoglobin (g/dl) (Mean SD) | 26,561 | 13.6 (1.7) | 12.4 (2.1) | <0.001 |
| White blood count (x10^9^/L) (Median IQR) | 26,561 | 7.2 (5.8-9.3) | 8.3 (6.4-11.7) | <0.001 |
| Lymphocyte count (x10^9^/L) (Median IQR) | 26,561 | 1.7 (1.3-2.2) | 1.3 (0.9-1.8) | <0.001 |
| Neutrophil count (x10^9^/L) (Median IQR) | 26,561 | 4.4 (3.2-6.2) | 5.7 (4.0-9.1) | <0.001 |
| **Nutritional and Metabolic Markers** |  |  |  |  |
| Serum Albumin (g/L) (Mean SD) | 32,666 | 38.1 (5.4) | 33.6 (6.9) | <0.001 |
| Serum Calcium (mmol/L) (Mean SD) | 32,666 | 2.3 (0.1) | 2.2 (0.2) | <0.001 |
| Serum Phosphorus (mmol/L) (Mean SD) | 32,666 | 1.1 (0.2) | 1.3 (0.4) | <0.001 |
| Serum Sodium (mmol/L) (Mean SD) | 32,666 | 139.0 (2.8) | 138.7 (4.0) | <0.001 |
| Corrected Serum Sodium (mmol/L) (Mean SD) | 32,666 | 139.0 (2.9) | 138.5 (4.1) | <0.001 |
| Serum Potassium (mmol/L) (Mean SD) | 32,666 | 4.4 (0.5) | 4.4 (0.6) | <0.001 |
| **Lipid related Markers** | | | | |
| Total Cholesterol (mmol/L) (Mean SD) | 17,188 | 5.1 (1.1) | 4.8 (1.2) | <0.001 |
| Triglycerides (mmol/L) (Mean SD) | 14,302 | 1.4 (0.9) | 1.4 (0.7) | <0.001 |
| **Glycaemic markers** |  |  |  |  |
| Glucose (mmol/L)  (Median IQR) | 32,666 | 5.2 (4.7-6.0) | 5.9 (5.1-7.6) | <0.001 |
| **Markers of Liver function** |  |  |  |  |
| Alanine Alkaline phosphatase (IU/L) (Median IQR) | 31,599 | 67.0 (54.0-83.0) | 76.0 (60.0-97.0) | <0.001 |
| Alanine transaminase (IU/L) (Median IQR) | 31,131 | 23.0 (18.0-32.0) | 20.0 (16.0-29.0) | <0.001 |
| Gamma-glutamyltransferase (IU/L) (Median IQR) | 31,196 | 24.0 (17.0-39.0) | 27.0 (18.0-47.0) | <0.001 |
| Total bilirubin (µmol/L) (Median IQR) | 30,867 | 12.0 (9.0-16.0) | 13.0 (10.0-17.0) | <0.001 |

a Clinical setting at baseline refers to the location of patient when the laboratory test was conducted.

b eGFR: Estimated glomerular filtration rate (ml/min per 1.73 m^2^) was based on the Chronic Kidney Disease Collaborative (CKD-EPI). ^18^
