# Supplementary material for: Impact of serum sodium concentrations, and effect modifiers on mortality in the Irish Health System
Source: BMC Nephrol. 2023 Jul 6;24:203. doi: 10.1186/s12882-023-03251-w (PMC10324141; doi:10.1186/s12882-023-03251-w)
Supplement: Supplementary file 4 — Additional file 4: Supplementary Table 2. Baseline Demographic and Clinical Characteristics of Study Population by Clinical Setting at Baseline. [file 12882_2023_3251_MOESM4_ESM.docx]

**Supplementary Table 2. Baseline Demographic and Clinical Characteristics of Study Population by Clinical Setting at Baseline**

| **Variable** | **N** | **GP** | **ER** | **IP** | **OP** | **P-value** |
| --- | --- | --- | --- | --- | --- | --- |
| Observations n (%) | 32,636 | 15,293 (46.9) | 4,835 (14.8) | 7,543 (23.1) | 4,965 (15.2) |  |
| Mean Age at baseline (SD) | 32,666 | 53.8 (16.6) | 56.6 (20.9) | 61.9 (17.1) | 58.7 (18.5) | <0.001 |
| **Sex** |  |  |  |  |  |  |
| Women | 17,367 | 55.3 | 45.3 | 53.3 | 54.1 |  |
| Men | 15,299 | 44.7 | 54.7 | 46.7 | 45.9 | <0.001 |
| **Markers of kidney function** |  |  |  |  |  |  |
| Urea (mmol/L) (Median IQR) | 32,666 | 4.8 (3.8-5.9) | 5.2 (3.8-7.5) | 5.1 (3.8-7.2) | 4.9 (3.9-6.4) | <0.001 |
| Serum creatinine (umol/L) (Median IQR) | 32,666 | 76.0 (65.0-88.0) | 82.0 (67.0-105.0) | 78.0 (63.0-99.0) | 77.0 (65.0-92.0) | <0.001 |
| eGFR at baseline (ml/min/1.73m^2^) (Median IQR)^a^ | 32,666 | 88.1 (73.5-101.8) | 82.0 (56.6-102.0) | 81.2 (58.3-97.9) | 84.1 (65.3-100.0) | <0.001 |
| **Inflammatory Markers** |  |  |  |  |  |  |
| Haemoglobin (g/dl) (Mean SD) | 26,561 | 14.0 (1.4) | 13.4 (2.0) | 12.4 (2.1) | 13.4 (1.8) | <0.001 |
| White blood count (x10^9^/L) (Median IQR) | 26,561 | 6.6 (5.5-7.9) | 9.4 (7.3-12.6) | 8.1 (6.2-11.2) | 7.2 (5.8-9.2) | <0.001 |
| Lymphocyte count (x10^9^/L) (Median IQR) | 26,561 | 1.8 (1.5-2.2) | 1.5 (1.0-2.1) | 1.4 (0.9-1.9) | 1.7 (1.3-2.2) | <0.001 |
| Neutrophil count (x10^9^/L)(Median IQR) | 26,561 | 3.8 (3.0-4.9) | 6.8 (4.6-9.9) | 5.5 (3.8-8.7) | 4.4 (3.4-6.0) | <0.001 |
| **Nutritional and Metabolic Markers** |  |  |  |  |  |  |
| Serum Albumin (g/L) (Mean SD) | 32,666 | 39.8 (3.3) | 36.3 (5.8) | 32.4 (7.6) | 37.9 (4.6) | <0.001 |
| Serum Calcium (mmol/L) (Mean SD) | 32,666 | 2.3 (0.1) | 2.3 (0.2) | 2.2 (0.2) | 2.3 (0.1) | <0.001 |
| Serum Phosphorus (mmol/L) (Mean SD) | 32,666 | 1.2 (0.2) | 1.2 (0.3) | 1.2 (0.3) | 1.2 (0.2) | <0.001 |
| Serum Sodium (mmol/L) (Mean SD) | 32,666 | 139.5 (2.3) | 137.9 (3.8) | 138.6 (3.7) | 139.0 (2.8) | <0.001 |
| Corrected Serum Sodium (mmol/L) (Mean SD) | 32,666 | 139.4 (2.4) | 137.8 (3.9) | 138.5 (3.8) | 138.9 (2.8) | <0.001 |
| Serum Potassium (mmol/L) (Mean SD) | 32,666 | 4.5 (0.5) | 4.1 (0.6) | 4.2 (0.5) | 4.3 (0.4) | <0.001 |
| **Lipid related Markers** |  |  |  |  |  |  |
| Total Cholesterol (mmol/L) (Mean SD) | 17,188 | 5.1 (1.1) | 4.8 (1.3) | 4.7 (1.2) | 5.0 (1.2) | <0.001 |
| Triglycerides (mmol/L (Mean SD) | 14,302 | 1.4 (0.9) | 1.4 (0.9) | 1.3 (0.8) | 1.4 (0.9) | <0.001 |
| **Glycaemic markers** |  |  |  |  |  |  |
| Glucose (mmol/L) (Median IQR) | 32,666 | 5.0 (4.6-5.5) | 6.3 (5.5-7.7) | 5.6 (5.0-7.0) | 5.5 (5.0-6.3) | <0.001 |
| **Markers of Liver function** |  |  |  |  |  |  |
| Alanine Alkaline phosphatase (IU/L) (Median IQR) | 31,599 | 67.0 (55.0-81.0) | 71.0 (56.0-91.0) | 71.0 (55.0-92.0) | 68.0 (56.0-86.0) | <0.001 |
| Alanine transaminase (IU/L) (Median IQR) | 31,131 | 23.0 (18.0-31.0) | 23.0 (17.0-35.0) | 22.0 (16.0-33.0) | 22.0 (17.0-30.0) | <0.001 |
| Gamma-glutamyltransferase (IU/L) (Median IQR) | 31,196 | 24.0 (17.0-36.0) | 27.0 (17.0-51.0) | 28.0 (18.0-52.0) | 23.0 (16.0-38.0) | <0.001 |
| Total bilirubin (umol/L) (Median IQR) | 30,867 | 12.0 (9.0-15.0) | 13.0 (10.0-18.0) | 13.0 (10.0-18.0) | 12.0 (9.0-15.0) | <0.001 |

a eGFR: Estimated glomerular filtration rate (ml/min per 1.73 m^2^) was based on the Chronic Kidney Disease Collaborative (CKD-EPI). ^18^

GP: General Practice, ER: Emergency Department, IP: Inpatient departments, OP: Outpatient departments,
