# Supplementary material for: Impact of serum sodium concentrations, and effect modifiers on mortality in the Irish Health System
Source: BMC Nephrol. 2023 Jul 6;24:203. doi: 10.1186/s12882-023-03251-w (PMC10324141; doi:10.1186/s12882-023-03251-w)
Supplement: Supplementary file 5 — Additional file 5: Supplementary Table 3. Relationship of Serum Sodium with All-Cause Mortality in the Health System. [file 12882_2023_3251_MOESM5_ESM.docx]

**Relationship of Serum Sodium with All-Cause Mortality in the Health System**

|  | **<135 mmol/L** | **135-145 mmol/L** | **> 145 mmol/L** |
| --- | --- | --- | --- |
| Baseline Observations (N) | 2,503 | 29,908 | 255 |
| Person years | 8,265.11 | 136,125.35 | 771.08 |
| Deaths | 882 | 4,131 | 101 |
| Crude Mortality rate per 1,000 pyrs | 106.71 | 30.35 | 130.98 |
| Age adjusted Mortlaity rate per 1,000 pyrs* | 42.5 | 20.3 | 62.4 |
| **Hazard Ratio for all-cause mortality**  **(95% CI)** |  | **Reference Group** |  |
| Serum sodium concentration  (defined at baseline) | 1.31 (1.21-1.43) | 1.00 | 1.45 (1.17-1.79) |
| Serum sodium concentration  (time-dependent model) | 1.80 (1.67-1.94) | 1.00 | 2.43 (2.06-2.88) |

Final model was adjusted for demographic factors (baseline age, sex), clinical indicators (haemoglobin, estimated glomerular filtration rate, serum albumin, serum potassium, serum calcium,white blood cell count, alanine aminotransferase, alkaline phosphatase), and clinical setting at baseline.

* Estimates are adjusted to mean age of 56.9 years
