# Supplementary material for: Impact of serum sodium concentrations, and effect modifiers on mortality in the Irish Health System
Source: BMC Nephrol. 2023 Jul 6;24:203. doi: 10.1186/s12882-023-03251-w (PMC10324141; doi:10.1186/s12882-023-03251-w)
Supplement: Supplementary file 6 — Additional file 6: Supplementary Table 4. Leading causes of death by baseline levels of Serum Sodium concentration. [file 12882_2023_3251_MOESM6_ESM.docx]

**Supplementary Table 4. Leading causes of death by baseline levels of serum sodium**

| **Mortality Type (%)** | | **Overall**  **(** N=5,114) | **<135**  **mmol/L** (N=882) | **135-145**  **mmol/L** (N=4,131) | **>145**  **mmol/L** (N=101) |
| --- | --- | --- | --- | --- | --- |
| **All Cardiovascular Diseases** | | **29.2** | **28.5** | **29.4** | **27.7** |
| Acute myocardial infarction | | 8.0 | 9.0 | 7.9 | 4.0 |
| Chronic ischemic heart disease | | 6.3 | 5.3 | 6.6 | 3.0 |
| Cerebral infarction | | 4.1 | 3.1 | 4.3 | 5.9 |
| Heart failure | | 2.2 | 1.9 | 2.2 | 4.0 |
| Atrial fibrillation and flutter | | 1.2 | 1.2 | 1.1 | 2.0 |
| Non-traumatic intracerebral haemorrhage | | 1.0 | 0.6 | 1.1 | - |
| Aortic aneurysm and dissection | | 0.8 | 0.3 | 0.8 | 2.0 |
| Other cerebrovascular diseases | | 0.6 | 0.5 | 0.7 | 2.0 |
| Complications and ill-defined descriptions of heart disease | | 0.5 | 0.8 | 0.5 | - |
| Non-rheumatic aortic valve disorders | | 0.5 | 0.8 | 0.4 | - |
| Other Cardiovascular diseases | | 4.1 | 5.0 | 3.8 | 5.0 |
|  | |  |  |  |  |
| **Malignant Neoplasms** | | **40.6** | **40.2** | **41.2** | **17.8** |
| Malignant neoplasm of bronchus and lung | | 7.1 | 8.7 | 6.9 | 1.0 |
| Malignant neoplasm of breast | | 3.9 | 3.2 | 4.2 | - |
| Malignant neoplasm of colon | | 3.2 | 3.3 | 3.2 | 3.0 |
| Malignant neoplasm of prostate | | 2.2 | 2.6 | 2.1 | 4.0 |
| Malignant neoplasm of ovary | | 1.6 | 1.8 | 1.6 | - |
| Malignant neoplasm of pancreas | | 1.5 | 1.5 | 1.5 | 2.0 |
| Multiple myeloma and malignant plasma cell neoplasms | | 1.3 | 0.8 | 1.4 | 1.0 |
| Myeloid leukaemia | | 1.3 | 1.9 | 1.2 | - |
| Malignant neoplasm of oesophagus | | 1.3 | 1.5 | 1.3 | - |
| Malignant neoplasm of stomach | | 1.3 | 1.5 | 1.3 | - |
| Other Neoplasms | | 15.8 | 13.5 | 16.6 | 6.9 |
|  | |  |  |  |  |
| **Noncardiovascular/Non-malignancy Diseases** | | **30.2** | **31.3** | **29.4** | **54.5** |
| Other chronic obstructive pulmonary disease | | 4.6 | 4.2 | 4.6 | 7.9 |
| Pneumonia, unspecified organism | | 3.6 | 3.7 | 3.6 | 4.0 |
| Unspecified dementia | | 1.1 | 0.6 | 1.2 | 5.0 |
| Other interstitial pulmonary diseases | | 1.0 | 0.7 | 1.0 | 2.0 |
| Unspecified diabetes mellitus | | 0.9 | 1.7 | 0.7 | - |
| Parkinson's disease | | 0.9 | 0.5 | 0.9 | 2.0 |
| Other respiratory disorders | | 0.8 | 0.7 | 0.8 | - |
| Chronic kidney disease (CKD) | | 0.7 | 0.5 | 0.8 | 1.0 |
| Alzheimer's disease | | 0.7 | 0.2 | 0.8 | - |
| Alcoholic liver disease | | 0.6 | 1.6 | 0.4 | 2.0 |
| Type 2 diabetes mellitus | | 0.6 | 0.8 | 0.6 | 1.0 |
| Other causes | | 11.8 | 13.7 | 11.1 | 21.8 |
| Unknown | 2.8 | | 2.5 | 2.8 | 7.9 |
