# Supplementary material for: Impact of serum sodium concentrations, and effect modifiers on mortality in the Irish Health System
Source: BMC Nephrol. 2023 Jul 6;24:203. doi: 10.1186/s12882-023-03251-w (PMC10324141; doi:10.1186/s12882-023-03251-w)
Supplement: Supplementary file 7 — Additional file 7:Supplementary Table 5. Age-adjusted mortality rates per 1,000 years of follow up by baseline serum sodium level*. Supplementary Table 6. Age-adjusted All-cause mortality rates per 1,000 years of follow up by baseline serum sodium level for specific age values*.Supplementary Table 7. Age adjusted mortality rates per 1,000 years of follow up by baseline serum sodium level* stratified by CKD subgroup.Supplementary Table 8. Age adjusted mortality rates per 1,000 years of follow up by baseline serum sodium level* stratified by clinical setting at baseline. [file 12882_2023_3251_MOESM7_ESM.docx]

**Supplementary Table 5. Age-adjusted mortality rates per 1,000 years of follow up by baseline serum sodium level***

| **Mortality Type** | **Serum Sodium Category** | | |
| --- | --- | --- | --- |
|  | **<135mmol/L** | **135-145 mmol/L** | **>145mmol/L** |
| All-cause | 42.5 (39.3-45.9) | 20.3 (19.5-21.2) | 62.4 (51.1-76.1) |
| Cardiovascular | 5.1 (4.3-6.0) | 3.1 (2.8-3.5) | 7.7 (5.2-11.3) |
| Malignancy | 26.2 (23.3-29.3) | 10.9 (10.3-11.5) | 16.2 (10.2-25.7) |
| Non cardiovascular/Non-Malignancy | 12.1 (10.5-14.0) | 5.6 (5.2-6.1) | 31.2 (23.7-41.1) |

*Estimates are adjusted to mean age of 56.9 years

**Supplementary Table 6 Age-adjusted All-cause mortality rates per 1,000 years of follow up by baseline serum sodium level***

|  | **Serum Sodium Category** | | |
| --- | --- | --- | --- |
|  | **<135mmol/L** | **135-145 mmol/L** | **>145mmol/L** |
| Age 40 | 14.4 (13.0-16.0) | 6.9 (6.4-7.4) | 21.2 (17.2-26.1) |
| Age 50 | 27.3 (25.1-29.8) | 13.1 (12.4-13.8) | 40.1 (32.8-49.1) |
| Age 60 | 51.8 (48.1-55.8) | 24.8 (23.9-25.7) | 76.0 (62.4-92.7) |
| Age 70 | 98.2 (91.8-105.0) | 47.0 (45.6-48.5) | 144.1 (118.5-175.2) |
| Age 80 | 186.0 (174.1-198.8) | 89.1 (85.9-92.4) | 273.1 (224.7-332.0) |

*Estimates are adjusted to age value as shown

**Supplementary Table 7. Age adjusted mortality rates per 1,000 years of follow up by baseline serum sodium level* stratified by CKD subgroup**

|  | | **Serum Sodium Category** | | | | | |
| --- | --- | --- | --- | --- | --- | --- | --- |
|  | | **<135mmol/L** | | **135-145 mmol/L** | | **>145mmol/L** | |
| **eGFR <60ml/min/1.72m^2^** | |  | |  | |  | |
| All-cause | | 65.9 (57.2-76.0) | | 41.6 (37.5-46.2) | | 96.0 (73.2-125.8) | |
| Cardiovascular | | 12.5 (9.6-16.1) | | 9.1 (7.5-11.1) | | 16.9 (10.4-27.5) | |
| Malignancy | | 28.6 (22.4-36.6) | | 17.5 (14.8-20.8) | | 30.8 (17.3-55.1) | |
| Non cardiovascular/Non-Malignancy | | 28.7 (22.8-36.0) | | 16.1 (13.6-19.1) | | 52.2 (35.1-77.5) | |
| **eGFR >=60ml/min/1.72m^2^** | |  | |  | |  | |
| All-cause | | 46.4 (42.1-51.2) | | 17.8 (17.0-18.7) | | 65.0 (47.9-88.1) | |
| Cardiovascular | | 5.0 (4.0-6.3) | | 2.4 (2.1-2.7) | | 7.7 (3.9-14.9) | |
| Malignancy | | 29.4 (25.7-33.5) | | 10.4 (9.8-11.1) | | 10.8 (4.9-24.1) | |
| Non cardiovascular/Non-Malignancy | | 11.5 (9.5-13.9) | | 4.4 (4.1-4.9) | | 39.4 (26.9-57.8) | |

*Estimates are adjusted to mean age of 56.9 years

**Supplementary Table 8. Age adjusted mortality rates per 1,000 years of follow up by baseline serum sodium level* stratified by clinical setting at baseline**

|  | | **Serum Sodium Category** | | | | | |
| --- | --- | --- | --- | --- | --- | --- | --- |
|  | | **<135mmol/L** | | **135-145 mmol/L** | | **>145mmol/L** | |
| **Inpatient** | |  | |  | |  | |
| All-cause | | 114.9 (103.7-127.3) | | 65.3 (61.8-68.9) | | 139.0 (109.2-176.9) | |
| Cardiovascular | | 10.5 (8.1-13.7) | | 7.1 (5.9-8.4) | | 13.6 (8.1-22.8) | |
| Malignancy | | 77.8 (68.0-88.9) | | 39.6 (37.0-42.3) | | 25.8 (13.9-48.0) | |
| Non cardiovascular/Non-Malignancy | | 24.3 (19.7-30.0) | | 15.1 (13.4-16.9) | | 71.3 (51.9-97.9) | |
| **Emergency Department** | |  | |  | |  | |
| All-cause | | 52.3 (45.1-60.7) | | 38.4 (34.7-42.5) | | 120.5 (78.5-185.0) | |
| Cardiovascular | | 11.2 (8.5-14.9) | | 9.6 (7.8-11.9) | | 22.6 (10.5-48.7) | |
| Malignancy | | 13.7 (10.1-18.5) | | 11.0 (9.2-13.3) | | 27.8 (10.3-74.9) | |
| Non cardiovascular/Non-Malignancy | | 28.0 (22.5-34.8) | | 16.7 (14.4-19.3) | | 74.0 (40.4-135.3) | |
| **General Practice** | |  | |  | |  | |
| All-cause | | 9.8 (7.9-12.3) | | 5.9 (5.4-6.6) | | 9.9 (4.7-20.8) | |
| Cardiovascular | | 1.9 (1.3-2.9) | | 1.5 (1.2-1.9) | | 2.0 (0.5-7.9) | |
| Malignancy | | 5.6 (4.0-8.0) | | 2.7 (2.3-3.1) | | 7.5 (2.8-20.1) | |
| Non cardiovascular/Non-Malignancy | | 2.8 (1.9-4.3) | | 1.7 (1.4-2.1) | | 1.4 (0.2-9.9) | |
| **Outpatient** | |  | |  | |  | |
| All-cause | | 21.0 (16.2-27.2) | | 14.8 (12.9-17.1) | | 30.1 (11.2-81.0) | |
| Cardiovascular | | 3.0 (1.8-5.0) | | 1.9 (1.3-2.8) | | 9.3 (2.9-30.1) | |
| Malignancy | | 12.8 (8.5-19.2) | | 9.1 (7.7-10.9) | | - | |
| Non cardiovascular/Non-Malignancy | | 4.3 (2.5-7.2) | | 3.6 (2.7-4.8) | | 6.5 (0.9-46.6) | |

*Estimates are adjusted to mean age of 56.9 years
